# An information intervention in India reduces consumption of arsenic-contaminated drinking water and has small effects on health

**DOI:** 10.1093/pnasnexus/pgag093

**Published:** 2026-03-27

**Authors:** Shambhavi Priyam, Daniel Salicath, Matthias Sutter

**Affiliations:** The World Bank, Washington, DC 20433, USA; Norwegian Labor and Welfare Administration, 0607 Oslo, Norway; Max Planck Institute for Research on Collective Goods, 53113 Bonn, Germany; Department of Economics, University of Cologne, 50935 Cologne, Germany; Department of Public Finance, University of Innsbruck, 6020 Innsbruck, Austria

## Abstract

Contamination of drinking water with toxic arsenic affects more than 170 million people worldwide. Because arsenic is tasteless, colorless, and odorless when mixed with water, almost all people consume it unknowingly and suffer from health impairments (like skin-related diseases or cancer). Running a randomized controlled trial with 2,334 Indian households, we show that providing inexpensive information on how to reduce exposure to arsenic contamination in drinking water has significant effects even 1 year after the intervention. First, it generates knowledge about arsenic's harmful effects. Second, it improves drinking water treatment practices. Third, treated households are between 2.7 and 9.3 percentage points more likely to consume safe drinking water. We also find suggestive evidence for improvements with respect to stomach-related health issues. With costs of <2 euros per disability-adjusted life year averted, our intervention is very cost-effective.

Significance Statement We present the results of a randomized controlled trial with 2,334 households in India and show that the consumption of arsenic-contaminated drinking water can be reduced. Given that more than 170 million people's health is seriously threatened by arsenic contamination, we study how to reduce exposure to arsenic contamination in drinking water with inexpensive measures. Our intervention is built on providing information about arsenic and how to improve safe drinking water treatment practices. We find that treated households are between 2.7 and 9.3 percentage points more likely to consume safe drinking water (according to the World Health Organization's threshold). With costs of <2 euros per disability-adjusted life year averted, our intervention is very cost-effective.

## Introduction

Arsenic-contaminated groundwater is consumed by more than 170 million people worldwide and has severe health consequences (1–5). Arsenic is a toxic, carcinogenic metallic element that has both negative short-term (like skin-related diseases) and long-term health consequences (such as cancer). It also impairs cognitive abilities, and combined with deteriorated health conditions, this has indirect effects on occupation, entrepreneurship, and income (6).

A major problem identifying arsenic contamination of drinking water is that it is tasteless, colorless, and odorless when mixed in water, for which reason most people affected by its presence are not even aware of it. This also holds true for the basin of the River Ganges in India and Bangladesh, where arsenic contamination is particularly pronounced, as arsenic deposits are naturally present in underground aquifers in this area. Previous interventions to reduce arsenic-contaminated water consumption in these areas have mainly aimed to convince households to switch to newer tube wells by clearly marking safe wells (7). It has been argued that these massive well-switching efforts undertaken by the government of Bangladesh from 1998 onwards were one of the most successful public health information campaigns (8, 9). Yet, this approach has also had its limitations. First, switching tube wells is inconvenient for many households, in particular, the poorest households who often live relatively far away from safe water sources (4). Second, the emphasis on changing tube wells implies the risk of actually switching to tube wells with even higher quantities of arsenic since previously safe water sources might turn toxic if the depletion of aquifers taps into contaminated water as the groundwater levels drop (10). Technical solutions—like using advanced reverse osmosis filters to clean the drinking water from arsenic, digging much deeper tube wells, or buying bottled water—are too expensive as solutions, in particular for the most vulnerable of the poor.

For this reason, we ran a field experiment in the low-caste hamlets of 156 villages in the Indian state of Bihar with the goal of helping this vulnerable population to cope with arsenic-contaminated drinking water. More precisely, we ran an information campaign in 2,334 households by providing low-cost and low-effort alternatives that do not focus on switching tube wells but try to motivate people to adopt simpler, healthier water treatment practices to avoid or reduce the consumption of arsenic-contaminated drinking water (or also of bacterial contamination). Our field intervention follows recent work where information was delivered in an audiovisual format and was found to be an effective tool for behavioral change in domains unrelated to arsenic (11–13). We evaluate the effectiveness of our intervention 2 months and 1 year later to study short- and longer-term effects. A 1-y-long follow-up has been rare so far in this literature on information interventions (14, 15). A longer gestation period is, however, necessary to measure the persistence of both information and health impacts and to examine whether they might take time to manifest. We measure the level of arsenic in a household's drinking water in an objective way by using a field test kit. Additionally, we collect self-reported health data, the household's water treatment practices, and their knowledge about arsenic. This way we can assess whether our low-cost intervention changes knowledge, behavior, the safety of a household's drinking water, and the household members' (self-reported) health.

## Study site and design

We have conducted our study in Bihar, a state in the northern Indian plains, south of the Himalayan mountains. Given the number of rivers that flow through Bihar, it is a fertile and mineral-rich agricultural state. Despite this, Bihar is one of the least developed states in India. In recent years, there has been an increase in cancer incidence in Bihar, which has been attributed to the high levels of arsenic in the groundwater (16). Field studies have shown that arsenic concentrations are on the rise in the area, and >40% of all districts in Bihar are affected by it (17).

We ran our study in the district of Samastipur (and some adjoining regions), which is representative of Bihar with respect to sex ratio, population growth, and literacy rates. The data collection was approved by the Institutional Review Board (IRB) of the Max Planck Society, Germany, and the IRB of Krea University, India. Signed informed consent was obtained from all participants before the survey in the language of the participant. Enumerators explained the purpose of the study, assured participants of confidentiality, and emphasized that participation was voluntary. Participants were informed that they could decline to answer any question or terminate the interview at any time. A copy of the consent form and contact information of survey administrators were left with the participants.

We carried out our baseline survey (before the intervention) from 2019 December 24 to 2020 February 3. At that time, no other campaigns about arsenic were active in the area. Consequently, at baseline, 98.3% of all participants stated having no knowledge about arsenic (and there was no difference in knowledge across the three treatment groups introduced below). Within our study area, we randomly selected 156 villages that were situated near the bank of the river Ganges (but were at least 500 m apart from each other). In prehistoric times, the river carried arsenic down from the mountains, which then coagulated in the plains due to sedimentation. In this area, arsenic occurs frequently in tube wells between 50 and 200 ft (15 and 60 m) in depth. Tube wells shallower than 50 ft would be above the arsenic belt, but as the aquifers get depleted, tube wells must be dug deeper to maintain water supply. Tube wells deeper than 200 ft, while possible, require extra monetary investments, usually not affordable by the poorest.

We chose to focus on the low-caste hamlets within the selected villages, as this group remains the most economically vulnerable, having limited access to safe water and health infrastructure, limited education, and higher exposure to the problem of arsenic in the groundwater, with few means to tackle the problem, compared with the high-caste hamlets in the same areas. We defined the low-caste hamlet in a village as the area where members of the Scheduled Castes/Scheduled Tribes and Other Backward Castes communities reside. Caste membership was verified through interviews with local government leaders or teachers in the Anganwadis (government kindergartens).

The experiment followed a clustered randomization design where we first randomly selected villages from a district, and then randomly selected within each village 15 households in the afore-described area of low-caste hamlets to be part of the study. We ran a baseline survey in 2,334 households. Immediately after the baseline survey, we implemented our intervention. The following treatments were administered at the village level (after having randomly assigned villages to one specific treatment).

### Individual information

Here, household heads (or their spouse if heads were absent) were shown an 8-min-long audiovisual containing arsenic-related information (see https://www.youtube.com/watch?v=puUDJQJ6qEs). Additionally, we asked the household head for a glass of drinking water from their source or storage unit and then tested it for arsenic and informed the household head whether the water was safe (according to World Health Organization [WHO] recommendations). The audiovisual contained information about the dangers of consuming arsenic-contaminated water, common misconceptions, and effective solutions to mitigate the problem with minimal financial investments. The latter include, for example, resting water overnight for settling down of particles before drinking (the top part of the rested water), using treated surface water, changing to a safer water source (usually a tube well), etc. The contents of the audiovisual were based on information from the United Nations Children's Fund and the World Bank and approved by two local authorities, the Bihar State Pollution Control Board and the Mahavir Cancer Center, Patna.

### Group information

This treatment was, in principle, identical to the individual information treatment, but we gathered three randomly selected household heads within a village and showed the video to them on the premises of one of their households. The arsenic measurement was done individually at each of the three households, and the result was communicated only to the respective household head. This treatment variation was intended to check whether a delivery of information in groups may yield stronger outcomes (due potentially to more conversation going on about the topic when delivered in groups) than in the individual treatment, partly motivated by experimental evidence that groups make more rational decisions than individuals (18, 19).

### Control group

In the control group for this experiment, the household heads were individually shown an 8-min-long audiovisual unrelated to arsenic or water (but rather tiger conservation, a video released by the Press Information Bureau of India and the Wildlife Trust; see https://www.youtube.com/watch?v=wrcWNtCc6Dk). We also measured the arsenic level of a glass of drinking water from the source or storage but did not report the result of the water test to the household head.

The intervention was implemented by a total of 23 experienced enumerators who were always divided into teams of four, led by one supervisor. The supervisors monitored randomization, conducted checks, and supported enumerators when required. Daily monitoring, frequency checks, and controls were conducted to ensure high data quality.

Midline data collection took place 2 months after the intervention. Due to COVID-19, the collection of midline data had to be halted on 2020 March 17 when 1,260 field surveys had been completed. Data collection resumed using phone surveys instead for the remaining sample, starting 2020 March 30. Yet, the phone survey had to be shortened to fit into a 30-min phone conversation and, of course, we were unable to conduct arsenic tests of the primary water source over the phone. The endline data collection, including arsenic tests and a detailed survey, was conducted in the households 1 year after the baseline (2021 January 14 to February 24) to study the longer-term effects of our intervention.

As our main outcome variables, we used (i) knowledge about arsenic, (ii) healthy water treatment practices, (iii) safety of drinking water with respect to arsenic, and (iv) self-reported health outcomes. We measured arsenic knowledge by administering a battery of 10 arsenic-related questions with responses “true or false” (see the survey in the Supplementary Information [SI]). Healthy water treatment practices were self-reported. Changing to a safer water source, letting water rest overnight, etc., are categorized as healthy treatment. Unhealthy treatment of water consists of behaviors such as boiling water that contains arsenic (from tube wells), drinking untreated surface water, etc. (see Table S.8 for an overview of healthy and unhealthy treatments of water; note that boiling surface water—that does not contain any arsenic—is considered a healthy treatment as it reduces bacterial contamination, contrary to the negative effects of boiling water from tube wells). As a means of ensuring compliance with self-reported behavior, the enumerators had to observe and take note of evidence of any changes in practices. For example, if the respondent mentioned that they installed a new tube well, the new infrastructure had to be shown to the enumerator, or if the respondent mentioned that they rested the water overnight, the storage unit was checked by the enumerator. The safety of the drinking water was measured using the ITS Arsenic Econo-Quick field test kits, a validated toolkit used by the WHO and academics (4, 20). The drinking water was classified as safe if it had at most 10 μg of arsenic per liter (which is the safety threshold used by the WHO). Finally, health outcomes were self-reported. We asked for the health condition of household members, referring to skin problems like hyperkeratosis, melanosis, and Mees' lines, stomach problems like diarrhea or constipation, and more severe problems like lung, liver, or cancer issues. Additionally, we elicited mental health with the Patient Health Questionnaire 9 (PHQ-9) (21), as arsenic has been indicated to have ill effects on it (3, 22).

## Results

On average, respondents in our study were 40 years old; 57% of them were female (because many household heads, traditionally the husbands in India, were absent); they had 3.4 years of education (but only 44% could read and write); and 5.8 people lived in their household. Practically, all of them were Hindu and married. At baseline, only 4.3% of households had no arsenic at all in their primary drinking water. Yet, 34.3% consumed drinking water that is considered unsafe, as its arsenic level exceeded the WHO limit of 10 μg/L. The remaining 61.4% of households had between 1 and 10 μg of arsenic per liter, which is still considered safe by the WHO. We show in the upper part of Fig. S.2 the distribution of arsenic levels at baseline, separately for our three treatment groups and measured in milligrams per liter (meaning that everything up to 0.01 mg is considered safe; this covers the first two bars of each panel in Fig. S.2). Moreover, we show in Table S.1 that the demographic characteristics are balanced across treatments and in Table S.2 that attrition does not depend on the treatment group. The attrition rate was very low, ranging from 1.9% in the individual to 3.2% in the control (and 2.3% in the group; with no significant difference across treatments, however), with a total of 58 households lost from baseline to endline.

For the following analyses, we use block-level fixed effects (blocks are administrative clusters of villages). Table 1 shows our main results for knowledge, water treatment practices, and the safety of the drinking water. It presents OLS regression outcomes for differences in our main outcomes at baseline, midline, and endline, and we control for the baseline level of arsenic in each regression to account for preexisting differences across households. The main variables shown are interaction effects of treatment (individual or group intervention) and midline and endline, respectively. In column 1, we show the standardized score of our quiz about arsenic. In both treatments, the score is significantly larger than in the control group. Actually, the scores increase over time, as the coefficients are significantly larger after 1 year (endline) than after 2 months (midline). We also note that while the fraction of households having “any” knowledge about arsenic was extremely small at baseline (1.3% in the individual, 1.8% in the group, and 2.0% in the control), this fraction increased considerably until endline, reaching 86.4% in the individual, 76.4% in the group, and even 29.5% in the control (the latter increase might be due to having raised the household's awareness about arsenic being potentially an issue, given that we had measured it).

**Table 1:** Differences in main outcomes on knowledge, practices, and safety.

	(1)	(2)	(3)
	Knowledge score (standardized)	Treatment practices (relative frequency of recommended practices)	Unsafe water consumption (according to WHO)
Midline	0.116***	−0.012	0.055*
	(0.037)	(0.009)	(0.029)
Endline	0.569***	0.014	0.016
	(0.068)	(0.013)	(0.023)
Individual	−0.001	−0.005	0.059
	(0.014)	(0.005)	(0.048)
Group	−0.003	−0.008	0.064
	(0.012)	(0.005)	(0.045)
Individual × midline	0.313***	0.031**	−0.065
	(0.059)	(0.013)	(0.045)
Individual × endline	1.348***	0.060**	−0.073**
	(0.087)	(0.022)	(0.036)
Group × midline	0.277***	0.029**	−0.072
	(0.073)	(0.013)	(0.050)
Group × endline	1.083***	0.050**	−0.024
	(0.106)	(0.024)	(0.031)
FE	Yes	Yes	Yes
Controls	Yes	Yes	Yes
Mean in control at baseline	−0.56	0.032	0.28
Observations	6,785	6,785	5,819
*R* ^2^	0.491	0.108	0.249

The table presents OLS estimates for differences in main outcomes at baseline, midline, and endline for each respondent. Column (1) shows differences in the standardized arsenic knowledge score for respondents who answered a set of 10 questions about arsenic in groundwater. These responses were aggregated into a single arsenic knowledge score. Column (2) shows differences in reported changes in safe water treatment practices. Healthy practices include those recommended in the informational video, such as boiling or filtering surface water, resting tube well water, and using reverse osmosis filter or bottled water. Column (3) shows differences in unsafe water consumption for each household. Unsafe water consumption refers to the fraction of households drinking water with arsenic levels exceeding WHO guidelines (10 μg/L), as measured by field test kits in the primary drinking water source of each household. Note that in midline, we could only measure the arsenic in the drinking water of 1,260 households. The models control for baseline arsenic levels to account for preexisting differences across households and in columns 1 and 2 for the baseline level of knowledge and treatment practices, respectively. Fixed effects (FE) are at the block level (administrative cluster of villages), and SEs are clustered at the village level.

For comparison reasons, we state here the baseline average values per treatment:

Knowledge score: control = −0.56, individual = −0.58, group = −0.56 (standardized index).

Safe water treatment practices: control = 0.032, individual = 0.019, group = 0.018 (relative frequencies).

Unsafe water consumption: control = 0.28, individual = 0.39, group = 0.36 (relative frequencies of having >0.01 mg/L arsenic in drinking water).

Mean of arsenic level: control = 0.027 mg/L, individual = 0.027 mg/L, group = 0.027 mg/L.

Significance at ***P < 0.01, **P < 0.05, *P < 0.1.

Column 2 in Table 1 presents the effects of our intervention on water treatment practices in the households. We see that they improve significantly in both treatments and at midline and endline. Again, we note an increase in coefficients from midline to endline, suggesting that some habits need time to build up.^a^ We note that in the baseline only 2.3% of households applied safe water practices. This fraction is raised by about 6 percentage points to ∼8–9% at the endline. This is still a very small fraction, showing that there is large scope for further improvements, but despite this limitation, we see that our treatment intervention improved practices. From baseline to endline, we observe that 163 households (∼7% of our total sample) improved water treatment practices. In Table S.3, we show which treatment practices were applied in those households. The data show quite some variation. In the control and group treatments, boiling the water was the modal choice, while resting water was the most popular measure in the individual treatment. Despite being informed about having high levels of arsenic in their tube well, none of our households changed to another tube well. In Table S.4, we then show what determines whether households that did not apply any safe water treatment practice at baseline shifted to at least one (and perhaps more) safe water treatment practice at endline. There, we see that the individual treatment has a significantly positive impact. For the group treatment, we note a positive coefficient, which fails to be significant, however.

Turning to the safety of the household's drinking water, column 3 in Table 1 shows the treatment effects on the likelihood to have too much arsenic in a household's drinking water (i.e. >10 μg/L). In other words, it illustrates whether a household's drinking water is considered unsafe by the WHO. Here, we see significantly negative effects for the individual treatment, reducing the likelihood of unsafe water consumption by 9.3 percentage points in the short run of 2 months, and 7.5 percentage points over 1 year. Judged against a baseline rate of 34% of households with unsafe water, this reduction can be considered substantial. For the group treatment, we see a similarly large coefficient (of −8.9 percentage points) for midline and a smaller one (−2.7 percentage points) for endline. Both coefficients in the group treatment fail significance, however (the midline coefficient has a *P*-value of 0.12; recall that in midline we have fewer observations, however, because of the restrictions of the COVID-19 pandemic).

In Table S.5, we show what happens to the *P*-values in Table 1 in case of controlling for multiple hypothesis testing. For that, we apply Holm–Bonferroni and global Benjamini–Hochberg (BH) corrections to the Table 1 coefficients. We see that the significance of our treatments on knowledge and water treatment practices largely remains unchanged. The effect of the individual treatment at endline (“Individual × Endline”) on the likelihood to consume unsafe water remains (weakly) significant with the BH correction but loses significance with the more conservative Holm–Bonferroni correction. Overall, the results in case of multiple hypothesis testing look similar, even if not always identical, to the results shown in Table 1.

In the following, we can provide some further exploratory analyses by looking into heterogeneity effects. To start with, recall that 163 households improved water treatment practices from baseline to endline. We can compare the relative frequency of unsafe water consumption (with respect to the arsenic level) in these households to the relative frequency in households that did not improve their practices (by just applying the same practices in endline as in baseline). We see a relative fraction of unsafe water consumption of 0.281 in the households that improved water treatment practices, but a level of 0.346 in the other households. The estimated difference (two-sample t test, *P* = 0.098) is suggestive of lower unsafe water consumption among households that improved treatment practices. In the SI, we provide further heterogeneity analyses. In Table S.6, we start with an analysis based on the level of poverty in our sample. We use the possession of a so-called “ration card” as a proxy for household economic status, as it is issued by the Indian government based on income and eligibility for subsidized food and fuel. Thus, possession and type of ration card are often used as indicators of wealth and poverty (see https://nfsa.gov.in/portal/PDS_page for an explanation of the program; we focus on the Antyodaya Anna Yojana [AAY] ration card which targets the poor). The first three columns of Table S.6 report knowledge scores, treatment practices, and arsenic levels for households without a ration card (the relatively wealthier households), and columns 4–6, the same for households with a ration card (the poorer households). Comparing coefficients across the associated columns, we see by and large similar effects with regard to knowledge and treatment practices, but with respect to unsafe water consumption we note a difference between both samples. Here, we see significant improvements (i.e. negative signs indicating less unsafe water consumption) only for the poorer sample, but not for the richer sample.

Given the small sample size, we can also take only an exploratory look into how the group composition in the group treatment affects treatment effects. We can see from Table S.7 that groups with a higher proportion of low-income participants (measured by ration card ownership as explained above) are less likely to have unsafe water consumption, while the proportion of ration card holders has no effect on knowledge and treatment practices.

In Table 2, we present the main results on self-reported health outcomes at the household level. For the analysis, we refer to endline data only, given that about half of the midline surveys had to be shortened due to COVID-19 (see above) and particularly also because health effects can be expected to need probably more time (than 2 months to midline) to show up. As dependent variables, we use dummy variables for whether any stomach-related issue, depression, or skin-related issues have been reported in a particular household (see table notes for definitions). In the first two columns, we see negative treatment effects throughout (meaning less stomach issues and less depression), but the coefficients are only significant for the group treatment for stomach issues. We take this as only suggestive evidence of some mild treatment effects on health outcomes (which may not only have been driven by a reduction of arsenic-contaminated water but also partly by boiling surface water and thus reducing bacterial contamination).

**Table 2:** Health outcomes at endline.

	(1)	(2)	(3)
	Dummy stomach issues	Dummy depression	Dummy skin issues
Individual × endline	−0.042	−0.042	0.025
	(0.036)	(0.043)	(0.030)
Group × endline	−0.093**	−0.010	−0.006
	(0.033)	(0.041)	(0.026)
FE	Yes	Yes	Yes
Controls	Yes	Yes	Yes
Observations	2,276	2,276	2,276
R^2^	0.050	0.013	0.089
Control mean	0.767	0.373	0.140

The table reports OLS estimates of treatment effects on endline health outcomes relative to the control group. In column (1), we report whether any stomach-related issues (like gastritis or constipation) were reported for any member of the household. Column (2) reports whether at least one household member was classified as being depressive according to the PHQ-9 survey. Column (3) reports any skin-related issues in the household (like skin irritations, melanosis, and hyperkeratosis). All specifications include household-level baseline controls and changes in knowledge between baseline and endline. The models control for baseline arsenic levels to account for preexisting differences across households. Block fixed effects (administrative clusters of villages) are included, and robust SEs are clustered at the village level. Significance levels: **P < 0.05.

Before concluding, we can also provide a back-of-the-envelope calculation of the cost-effectiveness of our interventions. We follow earlier approaches (23, 24) and take the estimated costs of daily labor of implementing the treatment and base the productivity of implementation as per the data in our study. For the individual treatment, this cost is 1.2 euros, and for the group treatment, it is 0.81 euros per household. Using reductions in unsafe water consumption over 1 year, we estimate the cost per DALY (disability-adjusted life year) averted to be ∼0.89 euros for the individual treatment and 1.84 euros for the group treatment.^b^ Recall, however, that the decrease in unsafe water consumption was not significant in the group treatment, for which reason the latter value should be taken with caution. Still, the cost-effectiveness seems to be very high in general, particularly so in the individual treatment, as the WHO (25) recommends classifying interventions in India as highly cost-effective if their costs align with the country's GDP per capita (of ∼1,855 euros in 2021).

Overall, our field experiment suggests that there are inexpensive ways to help people to cope better with arsenic-contaminated drinking water, which can then also improve people's health. While the intervention was designed to reduce exposure to arsenic contamination, the observed improvements in health outcomes and safe water practices may also reflect incidental reductions in fecal-bacterial contamination or behavioral changes not directly targeted by the intervention. This underscores both the potential for water quality interventions to generate broader benefits than initially anticipated and the value of incorporating a wider set of water quality measurements in future studies. Our information intervention has had effects even 1 year after the intervention, which raises the interesting question for future research whether the effects may persist even several years after the intervention. Moreover, another feature worthy of further investigation would be to collect health data that are verified by health practitioners to get more objective measures of longer-term effects of our intervention on health. We believe that measuring the arsenic level of a household's drinking water with the field test kit is an important step for objectively measuring treatment effects, but having even better health data would probably help to identify the effects even more carefully in the future.

## Supplementary Material

pgag093_Supplementary_Data

## Data Availability

The data used for this analysis, including survey data without personal identifiers, protocols, survey instruments, and code, used have been deposited in Edmond—Open Research Data Repository of the Max Planck Society at https://doi.org/10.17617/3.LPX8BT.

## Notes

a Recall that in midline, we were interviewing about 46% of our sample via telephone (in reaction to the COVID-19 shutdown). While we could not measure the level of arsenic in these households’ drinking water, we can compare their knowledge and treatment practices with those households which were interviewed face-to-face. We believe it is noteworthy that the households with telephone interviews reported always better water treatment practices than households with face-to-face interviews, and in two out of three treatments this difference is even significant (Table S.9). It might be the case that over the phone it is easier to report healthy treatment practices (which supports our inclination to give more weight to endline data than midline data). For knowledge scores, we do not see such a pattern.

b For these calculations, we take the average costs of an optimistic scenario (in which subjects live 10 more years after cancer detection) and a pessimistic scenario (in which they live only for one more year after cancer detection). We estimate the average expected cost of exposure averted to be 16 euros for the individual treatment and 33 euros for the group treatment, based on reductions in unsafe water consumption (7.5 percentage points for individual treatment and 2.7 percentage points for group treatment in the course of 1 year). We assume that arsenic-linked cancers are often detected at an advanced stage, with an average age of detection of 44.5 years in Bihar (16) and a life expectancy of 69.1 years. Using reductions in unsafe water consumption over one year, we estimate the cost per DALY (disability-adjusted life year) averted to be between 0.68 and 1.1 euros for the individual treatment and between 1.4 and 2.28 euros for the group treatment.
