# Supplementary material for: An information intervention in India reduces consumption of arsenic-contaminated drinking water and has small effects on health
Source: PNAS Nexus. 2026 Mar 27;5(4):pgag093. doi: 10.1093/pnasnexus/pgag093 (PMC13082228; doi:10.1093/pnasnexus/pgag093)
Supplement: pgag093_Supplementary_Data [file pgag093_supplementary_data.pdf]

Supplementary Information (SI)

An information-intervention in India reduces consumption of arsenic-contaminated drinking water and has small effects on health

Shambhavi Priyam, Daniel Salicath and Matthias Sutter

Table S.1: Balance tests

	Control		Individual		Group		Pairwise tests		
	(1)	(2)	(3)	(4)	(5)	(6)	(7)	(8)	(9)
	N. obs.	Control mean	N. obs.	Individual mean	N. obs.	Group mean	(2) vs . (4) *p*-value	(2) vs . (6) *p*-value	(4) vs . (6) *p*-value
Unsafe water (=1)	760	0.28	792	0.39	782	0.36	0.733	0.305	0.619
Baseline arsenic level	760	0.03	792	0.03	782	0.03	0.354	0.933	0.326
Age	753	39.69	781	40.67	770	40.04	0.105	0.636	0.315
Female (=1)	753	0.57	781	0.55	770	0.60	0.258	0.355	0.050*
Years of education	753	3.65	781	3.41	770	3.03	0.822	0.150	0.122
Low caste (=1)	760	0.81	792	0.80	782	0.86	0.809	0.262	0.289
Respondent saves (=1)	760	0.59	792	0.67	782	0.65	0.126	0.079*	0.716
Number of people with savings	760	1.42	792	1.58	782	1.48	0.452	0.579	0.512
Wealth index (0-15)	760	8.27	792	8.33	782	7.80	0.373	0.088*	0.010**
Knowledge score	760	0.02	792	-0.04	782	0.02	0.370	0.719	0.399
Treatment practices	760	0.03	792	0.02	780	0.02	0.254	0.073*	0.825

Notes: The table reports group means of key baseline characteristics, including standardized arsenic knowledge score and safe water treatment practices. Unsafe water represents the fraction of households drinking water with arsenic levels exceeding WHO guidelines (ten micrograms per liter). Baseline arsenic level, unsafe water, standardized knowledge score and safe water treatment practices are all based on household-level measurements or survey responses. The standardized knowledge score is aggregated from ten survey questions about arsenic in groundwater. Safe water treatment practices include behaviors promoted in the informational video, such as boiling or filtering surface water, resting tubewell water, and using reverse-osmosis filters or bottled water. Stars indicate whether the differences between groups are statistically significant. Of the multiple comparisons across baseline outcomes, only one difference is significant at the 5% level (without adjusting for multiple testing), which is roughly what would be expected by chance. At the 10% level, three differences are significant, again consistent with chance expectations. Significance is indicated as * p<0.10, ** p<0.05, *** p<0.01.

Table S.2: Attrition

	(1)<br>Midline	(2)<br>Endline
Individual (=1)	-0.173	-0.417
	(0.279)	(0.308)
Group (=1)	-0.214	-0.289
	(0.282)	(0.284)
Unsafe water (=1)	-0.235	-0.764*
	(0.241)	(0.410)
Baseline arsenic level	1.694	1.282
	(1.516)	(2.390)
Age	0.009	-0.016
	(0.008)	(0.015)
Female (=1)	-0.384*	0.079
	(0.231)	(0.297)
Years of education	-0.042*	-0.006
	(0.024)	(0.029)
Low Caste (=1)	-0.141	0.109
	(0.237)	(0.377)
Respondent saves (=1)	-0.108	-0.625*
	(0.219)	(0.320)
Number of people with savings	-0.059	-0.037
	(0.075)	(0.147)
Wealth index (0-15)	-0.140***	-0.078
	(0.040)	(0.052)
N.obs.	2,273	2,025

Notes: Logit estimates for differences in baseline characteristics for the attrited sample at midline and endline (pooling control and treatment groups). No treatment-specific attrition was observed, with significant differences found in only a few outcomes. At endline, 248 observations were excluded from the analysis, as there was no attrition in one of the blocks. Fixed effects (FE) are at the block level (administrative cluster of villages) and standard errors are clustered at the village level.

Significance at $p<0.10$, ** $p<0.05$, *** $p<0.01$.

Table S.3: Water treatment practices for those 163 households who improved from baseline and endline (in percent as relative frequencies)

	Boil Water Safely (if surface water)	Filter Candle	Filter with RO	Filter with Cloth	Buy Bottled Water	Rests Water Safely
Control	46.4	0.0	3.6	14.3	3.6	32.1
Individual	15.7	2.0	9.8	15.7	5.9	52.9
Group	41.5	0.0	0.0	9.8	12.2	36.6

Notes: The table shows the percentage breakdown of water treatment practices for those households that improved between baseline and endline. Overall improvement in safe water practices: Individual: 6.6 percentage points, Group: 5.4 percentage points, Control: 3.8 percentage points.

Table S.4: Water treatment practices, conditional on treatment

	(1)<br>Dummy for applying any safe water treatment practices
Individual (=1)	0.758**
	(0.301)
Group (=1)	0.420
	(0.327)
Unsafe water (=1)	0.215
	(0.304)
Baseline arsenic level	-5.767
	(3.648)
Age	-0.014*
	(0.008)
Female (=1)	0.034
	(0.225)
Years of education	-0.009
	(0.022)
Low Caste (=1)	0.120
	(0.284)
Respondent saves (=1)	0.123
	(0.231)
Number of people with savings	0.082
	(0.070)
Wealth index (0-15)	0.093*
	(0.047)
N.obs.	2,273

Notes: Logit estimates for applying any safe water treatment practices at endline (pooling control and treatment groups), conditional on not having applied any safe water treatment practice at baseline. Fixed effects (FE) are at the block level (administrative cluster of villages) and standard errors are clustered at the village level. Significance at $p<0.10$, ** $p<0.05$, *** $p<0.01$.

Table S.5. Multiple Hypothesis Testing for Table 1: Differences in main outcomes on knowledge, practices and safety

Individual × Midline

Hypothesis	*Treatment effect*	*Raw p*	*Holm-Bonferroni*	*BH (global FDR)*
Knowledge score	0.313	0.000	0.000	0.000
Treatment practices	0.031	0.019	0.133	0.038
Unsafe water consumption	-0.065	0.146	0.438	0.175

Individual × Endline

Hypothesis	*Treatment effect*	*Raw p*	*Holm-Bonferroni*	*BH (global FDR)*
Knowledge score	1.348	0.000	0.000	0.000
Treatment practices	0.060	0.009	0.072	0.022
Unsafe water consumption	-0.073	0.044	0.176	0.059

Group × Midline

Hypothesis	*Treatment effect*	*Raw p*	*Holm-Bonferroni*	*BH (global FDR)*
Knowledge score	0.277	0.000	0.000	0.000
Treatment practices	0.029	0.029	0.174	0.050
Unsafe water consumption	-0.072	0.156	0.312	0.170

Group × Endline

Hypothesis	*Treatment effect*	*Raw p*	*Holm-Bonferroni*	*BH (global FDR)*
Knowledge score	1.083	0.000	0.000	0.000
Treatment practices	0.050	0.035	0.175	0.053
Unsafe water consumption	-0.024	0.445	0.445	0.445

Notes: Coefficients are taken directly from the Table 1 regressions, which include the same controls, block fixed effects, and village-clustered standard errors. Raw p-values correspond to individual hypothesis tests and are reported without correction for multiple testing. Holm–Bonferroni adjusted p-values control the family-wise error rate across outcomes within each panel. BH (global FDR) adjusted p-values apply the Benjamini–Hochberg false discovery rate correction across all outcomes reported in the table.

Table S.6: Differences in main outcomes on knowledge, practices and safety by household ration card status

	(1)	(2)	(3)	(4)	(5)	(6)
	No rations card	No rations card	No rations card	Rations card	Rations card	Rations card
	Knowledge score	Treatment practices	Unsafe water consumption	Knowledge score	Treatment practices	Unsafe water consumption
Midline	0.098**	–0.018	0.074**	0.138**	–0.008	0.023
	(0.035)	(0.011)	(0.037)	(0.052)	(0.014)	(0.038)
Endline	0.698***	0.016	0.048	0.404***	0.007	–0.018
	(0.081)	(0.017)	(0.032)	(0.083)	(0.016)	(0.026)
Individual	–0.006	–0.016	0.032	–0.001	–0.002	0.097
	(0.023)	(0.013)	(0.056)	(0.026)	(0.012)	(0.054)
Group	–0.026	–0.022	0.060	0.027	–0.004	0.057
	(0.023)	(0.011)	(0.054)	(0.024)	(0.012)	(0.049)
Individual x Midline	0.354***	0.039**	–0.047	0.263***	0.016	–0.081
	(0.063)	(0.016)	(0.056)	(0.083)	(0.020)	(0.059)
Individual x Endline	1.196***	0.051**	–0.079*	1.542***	0.057*	–0.065
	(0.101)	(0.024)	(0.044)	(0.108)	(0.033)	(0.049)
Group x Midline	0.327***	0.039**	–0.080*	0.214**	0.016	–0.045
	(0.080)	(0.015)	(0.059)	(0.092)	(0.020)	(0.061)
Group x Endline	0.908***	0.068**	–0.044	1.308***	0.016	0.001
	(0.119)	(0.029)	(0.038)	(0.127)	(0.029)	(0.041)
FE	YES	YES	YES	YES	YES	YES
Controls	YES	YES	YES	YES	YES	YES
Observations	3,825	3,825	3,268	2,960	2,960	2,551
R-squared	0.452	0.021	0.234	0.518	0.023	0.290

Notes: The table presents OLS estimates of changes in main outcomes for two subsamples: columns (1)–(3) restrict the sample to households with no rations card, and columns (4)–(6) restrict the sample to ration card holders. Knowledge score (columns 1 and 4): standardized arsenic knowledge score for respondents who answered a set of ten questions about arsenic in groundwater. These responses were aggregated into a single arsenic knowledge score. Treatment practices (columns 2 and 5): reported changes in safe water treatment practices. Healthy practices include those recommended in the informational video, such as boiling or filtering surface water, resting tubewell water, and using reverse-osmosis (RO) filter or bottled water. Unsafe water consumption (columns 3 and 6): fraction of households drinking water with arsenic levels exceeding WHO guidelines (ten micrograms per liter), as measured by field test kits in the primary drinking water source of each household. Note that in midline we could only measure the arsenic in the drinking water of 1,260 households. Fixed effects (FE) are at the block level (administrative cluster of villages) and standard errors are clustered at the village level. Significance at *** p<0.01, ** p<0.05, * p<0.1.

Table S.7: Effects of group composition with a ration card

	(1)	(2)	(3)
	Knowledge score	Treatment practices	Unsafe water consumption
Proportion ration card	-0.062	0.016	-0.047*
	(0.053)	(0.014)	(0.026)
Midline	19.988	-5.074	14.810*
	(16.599)	(4.312)	(0.083)
Endline	21.244	-5.015	14.838*
	(16.594)	(4.309)	(0.083)
FE	YES	YES	YES
Controls	YES	YES	YES
Observations	2,268	2,268	1,914
R-squared	0.454	0.022	0.341

Notes: The table shows the effect of group composition with a ration card in the group treatment. Group composition is assumed based on the timing of when households completed the baseline survey. Column (1) shows differences in the standardized arsenic knowledge score for respondents who answered a set of ten questions about arsenic in groundwater. These responses were aggregated into a single arsenic knowledge score. Column (2) shows differences in reported changes in safe water treatment practices. Healthy practices include those recommended in the informational video, such as boiling or filtering surface water, resting tubewell water, and using reverse-osmosis (RO) filter or bottled water. Column (3) shows differences in unsafe water consumption for each household. Unsafe water consumption refers to the fraction of households drinking water with arsenic levels exceeding WHO guidelines (ten micrograms per liter), as measured by field test kits in the primary drinking water source of each household. Note that in midline we could only measure the arsenic in the drinking water of 1,260 households. The models control for baseline arsenic levels to account for pre-existing differences across households. Fixed effects (FE) are at the block level (administrative cluster of villages) and standard errors are clustered at the village level. Significance at *** p<0.01, ** p<0.05, * p<0.1.

Table S.8: Description of healthy water treatment practices

Water Source	Treatment					
	None	Boil	Filter Candle	Chlorine	Filter with RO	Filter with cloth
Pond/river/surface water		x	x	x	x	
Tubewell with As					x	
Tubewell without As	x	x	x	x	x	x
Well		x	x	x	x	
Waterfall/spring		x	x	x	x	
Rainwater		x	x	x	x	
Bottled water	x	x	x	x	x	x
Borewell withs As					x	
Borewell without As	x	x	x	x	x	x

Notes: Most of the water sources and treatments were in these categories. An “other" category was created for both treatment and source which was determined to be healthy on a case-by-case basis. x indicates healthy practice

Table S.9: Differences in outcomes by interview mode (Phone vs face-to-face (FTF)) at Midline

Mode x Treatment	(1)<br>Knowledge score	(2)<br>Treatment practices
Control – FTF	-0.456	0.012
Control – Phone	-0.428	0.039
Diff FTF−Phone	-0.028	-0.028*
	(0.041)	(0.011)
Individual – FTF	-0.089	0.031
Individual – Phone	-0.241	0.061
Diff FTF−Phone	0.152*	-0.030*
	(0.065)	(0.015)
Group – FTF	-0.211	0.026
Group – Phone	-0.124	0.053
Diff FTF−Phone	-0.087	-0.026
	(0.066)	(0.014)
Observations	2,178	2,178

Notes: The table reports means for face-to-face (FTF) and phone respondents at midline separately for each treatment. Column (1) shows differences in the standardized arsenic knowledge score for respondents who answered a set of ten questions about arsenic in groundwater. Column (2) shows differences in reported changes in safe water treatment practices. Healthy practices include those recommended in the informational video, such as boiling or filtering surface water, resting tubewell water, and using reverse-osmosis (RO) filter or bottled water. "Diff FTF−Phone" is the difference in means (FTF minus Phone) with standard errors in parentheses below. Positive values indicate that FTF respondents scored higher than phone respondents, negative values indicate the opposite. Significance at *** p<0.01, ** p<0.05, * p<0.1.
Please note that the knowledge score is standardized using the pooled sample mean and standard deviation; therefore, levels need not be centered at zero within a given survey wave.

Figure S.1: Map of treatment and control villages in Bihar, India

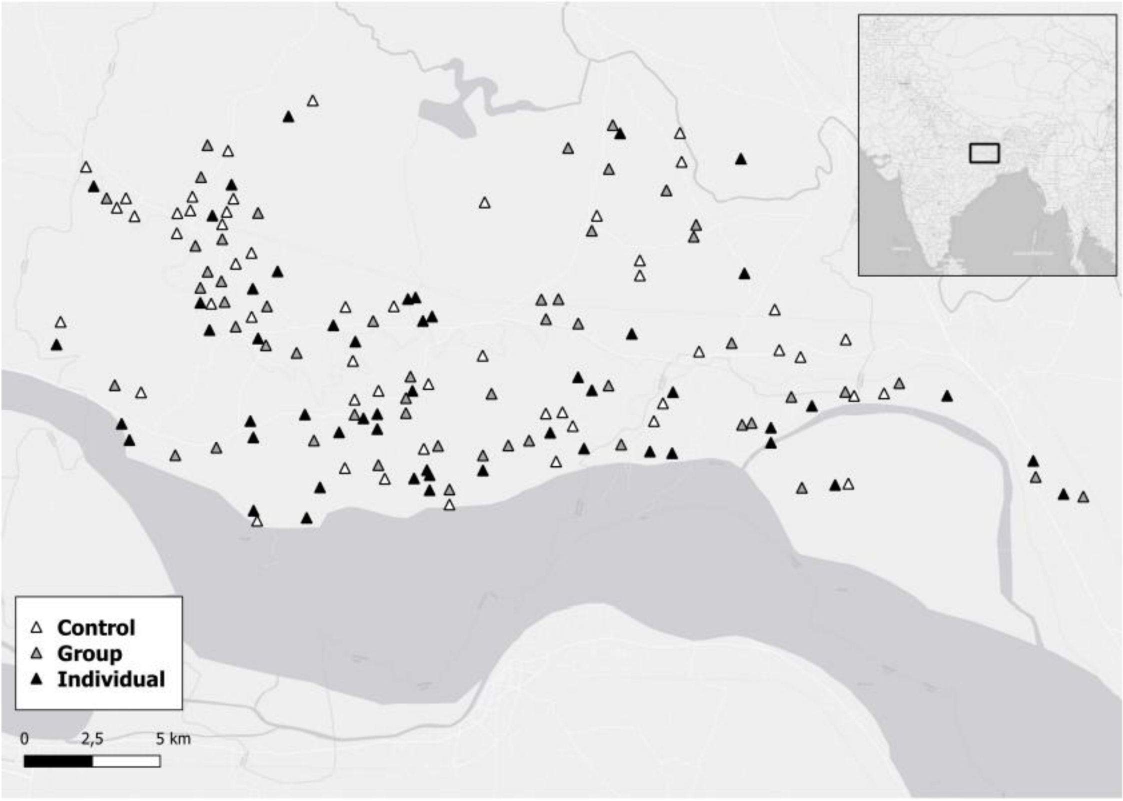

Figure S.2. Distribution of arsenic levels, conditional on treatment and separately for Baseline and Endline (measured in milligrams per liter)

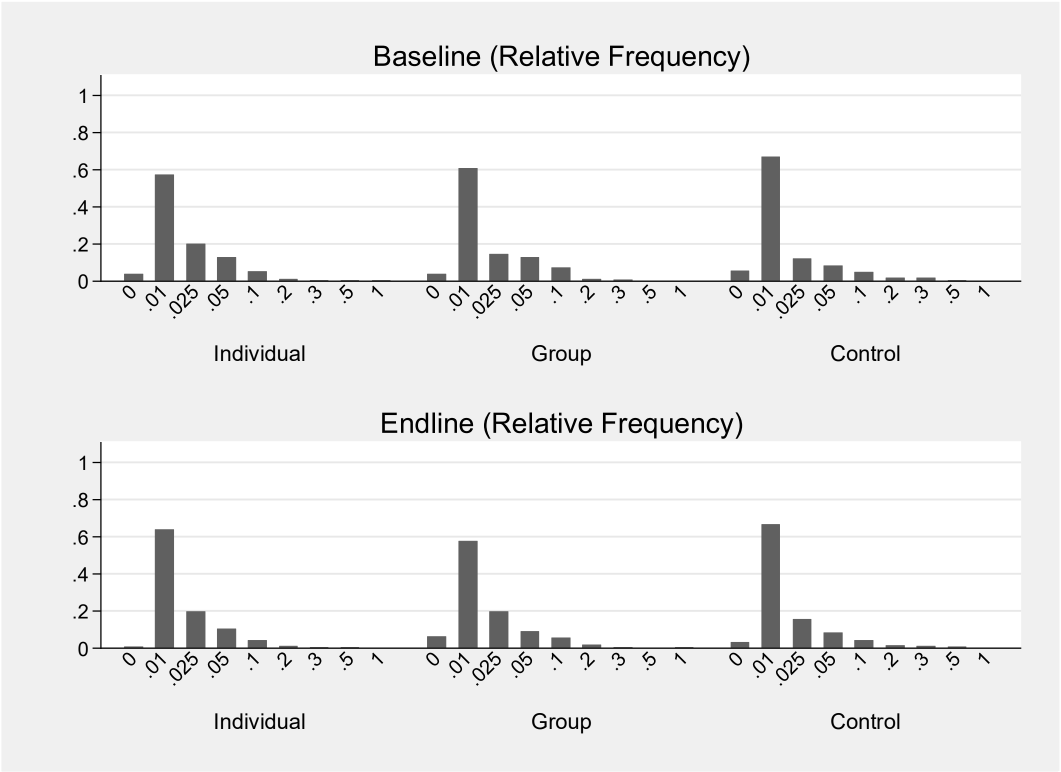

Household Survey

General Information

Name of respondent

Relationship of respondent to household head? O Self O Spouse

Is the household head male? O Yes O No

Religion [Hindu, Muslim, Christian, Sikh, Jain, Other]

Caste [Scheduled Caste, Scheduled Tribe, OBC, General, Other]

Household Roster

Member ID Relationship to HH Age Gender Primary, employment, Marital status, Education, Days of work/school missed due to illness/injury in past month, Arsenic symptoms, Healthcare treatment?, Smoker? Migrated in the last year? Diarrhea in past month?

Relationship to HH head	Employment	Arsenic symptoms
1. Self<br>2. Spouse<br>3. Own child<br>4. Step-child<br>5. Parent<br>6. Sibling<br>7. Grandparent<br>8. Grandchild<br>9. Cousin<br>10. Nephew/Niece<br>11. Son/dau-in-law<br>12. Bro/sis-in-law<br>13. Parent-in-law<br>14. Aunt/Uncle	1. Self-employed (agri)<br>2. Self-employed (non-agri)<br>3. Agricultural labor<br>4. Non-agriculture labor<br>5. Independent/skilled work<br>6. Own shop/business<br>7. Household work<br>8. Pension<br>9. Rental income<br>10. Regular wage/salary<br>11. Student<br>12. Does not work	1. Hyperkeratosis in palm and sole<br>2. Melanosis in palm and trunk<br>3. Other skin problem - irritation<br>4. Anemia<br>5. Gastritis<br>6. Liver problem<br>7. Constipation<br>8. Loss of appetite<br>9. Infertility<br>10. Irregular menstrual cycle<br>11. Asthma,or Bronchitis<br>12. Cancer<br>13. None

Arsenic knowledge

Arsenic is an element that occurs naturally. It is found in drinking water, food, and air. If you ingest more than a certain amount of arsenic, it is toxic for the human body. Now we will ask you some questions about arsenic. We only want to understand how much you know about it. You will not be getting a prize for this section, so please answer only what you know.

1. Do you have any knowledge about arsenic? Yes/No
2. Many people in Bihar die from drinking arsenic in water Yes/No/Don’t know
3. Arsenic in the drinking water is visible Yes/No/Don’t know
4. It’s possible to taste arsenic in drinking water Yes/No/Don’t know
5. Digging a deeper tubewell decreases arsenic exposure Yes/No/Don’t know
6. If you leave arsenic water overnight the top half become potable Yes/No/Don’t know
7. You should always boil water you use for drinking or cooking Yes/No/Don’t know
8. When you have arsenic in you tubewell, switching to another well is always a good option Yes/No/Don’t know
9. Tubewell water with arsenic is often healthier than treater

surface water Yes/No/Don't know
10. Using arsenic water can cause cancer and lung diseases Yes/No/Don't know
11. Skin diseases caused by arsenic are contagious Yes/No/Don't know

Water usage
1. Have you ever seen a public or NGO campaign about arsenic in the groundwater? Yes/No/Not sure
2. Does the household own a tubewell or is there one within the compound? Yes/No/Not sure
3. How deep is the tubewell? (feet)
4. Has the tubewell been tested for arsenic? Yes/No/Not sure
5. Was it considered safe? Yes/No
6. Cooking and Drinking water *(same questions asked for each)*
a. What is your primary source of cooking water?
[Pond/river/surface water, Tubewell, Piped water, Well, Waterfall/spring, Rain water, Buy bottled water, Borewell, Other]
b. Has you HH changed primary cooking water source in the past 2 years? Yes/No/Not sure
c. Do you know if there is arsenic in the primary cooking water? [It is arsenic free; Yes, it has arsenic; Don't know]
d. Do you know if there is iron in the primary cooking water? [It is iron free; Yes, it has iron; Don't know]
e. Has primary cooking water been tested for arsenic? Yes/No/Not sure
f. Do you treat the cooking water in any way before using it? [No, Boil it, Filter with candle filter, Chlorinate, Filter with RO, Filter with cloth, Other]
g. How frequently do you do this treatment? [Always, Usually, Sometimes, Rarely]
h. Do you think your cooking water is safe? Yes/No/Not sure
i. If you had an answer for the last question, how certain are you
of this? [Not certain, Somewhat certain, Very certain]
j. Why do you think so? [Seen the test/label (red), Everyone knows it, Someone told me, Close to other such sources, Tubewell is deep, From experience, Water pot turns red, Don't remember, Other]
k. Color of your cooking water Clear/Brown/Yellow
l. How far is your primary cooking water source?
7. Do you use the same drinking source for irrigation and animal feeding? Yes/No/Not sure/Other
8. Would you consider getting an RO if you don't have one installed? Yes/No/Not sure/Has RO
9. Do you know anyone that has arsenic in their primary drinking water? Yes/No/Not sure
10. Do you know of anyone who has become sick due to dirty drinking water? Yes/No/Not sure
11. Do you know anyone who has got a skin disease due to drinking water? Yes/No/Not sure
12. How willing are you to change your primary drinking water source? [Not willing, Somewhat willing, Very willing]
13. How much water does the household use on an everyday basis? [0-10 liters, 11-20 liters, 21-30 liters, 31-40 liters, 41-50 liters, Above 50 liters
14. Do you know of a communal arsenic free water source in your village? Yes/No

Wealth/Asset Index
Now I will ask you about a few things, and you have to tell me whether you have them in your house or not.
1. Mobile?
2. Electricity?
3. Radio?
4. Television?
5. Fan?
6. Mosquito net?
7. Bicycle?
8. Motorcycle/scooter?
9. Car?
10. Pair of shoes for everyone (sandals for women)?
11. Chair?
12. Gas stove?
13. Pressure cooker?

14. Pacca kitchen?
15. Pacca bathroom?
16. Antodaya card?

Savings
Does {Member ID} currently have an MFI loan?(Yes/No)
Amount of MFI Loan
Saves in other ways?
(Yes/No)
Savings mechanism [Post bank, Village bank, SHG, Friend, Family member, Coworker, Employer, Commercial bank, Microfinance institution, At home, Moneylender, Other]

Mental Health
A. *Patient Health Questionnaire (PHQ-9)* Over the last two weeks, how often have you been bothered by any of the following problems:
Options: Not all days/Several days/ More than half the days/ Nearly everyday
•Little interest or pleasure in doing things?
•Feeling down, depressed, or hopeless?
•Trouble falling or staying asleep, or sleeping too much?
•Feeling tired or having little energy?
•Poor appetite or overeating?
•Feeling bad about yourself - or that you are a failure or have let yourself or your family down?
•Trouble concentrating on things, such as reading the newspaper or watching television?
•Moving or speaking so slowly that other people could have noticed? Or the opposite - being so fidgety or restless that you have been moving around a lot more than usual?
•Thoughts that you would be better off dead, or of hurting yourself in some way?

B. *Loneliness*
Options: Yes/No/Don't know
During the past few weeks, did you ever feel very lonely or remote from other people?

Volume of conversation
1. In the past 30 days, did you have conversations about arsenic in the groundwater? OYes ONo
2. In how many of them did you approach other people so that you could discuss arsenic in the groundwater?
3. In how many of them did someone else seek you out so that you could discuss arsenic in groundwater?

Post-video

Control group
The audio visual is shown after the respondent has completed the questionnaire. After watching the audio-visual, the respondent answers the following three questions:
1. How much did you like the video?
0 = Did not like at all Liked very much = 10
0 1 2 3 4 5 6 7 8 9 10
2. Did you relate to the video?
0 = Did not relate at all Related very much = 10
0 1 2 3 4 5 6 7 8 9 10
3. Did you learn anything new from the video?
0 = Did not learn anything new Learned a lot of new things = 10
0 1 2 3 4 5 6 7 8 9 10

Treatment groups
The audio visual is shown after the respondent has completed the questionnaire. After watching the audio-visual, the respondent answers the following three questions:
1. How much did you like the video?
0 = Did not like at all Liked very much = 10

0 1 2 3 4 5 6 7 8 9 10
2. Did you relate to the video?
0 = Did not relate at all Related very much = 10
0 1 2 3 4 5 6 7 8 9 10
3. Did you learn anything new from the video?
0 = Did not learn anything new Learned a lot of new things = 10
0 1 2 3 4 5 6 7 8 9 10

Drinking water arsenic test
Arsenic tests are conducted following the standard procedure provided by ITS Econo-Quick test kit.
Arsenic quantity measured: [0 | 0.01 | 0.025 | 0.050 | 0.1 | 0.2 | 0.3 | 0.5 | 1]
Conclusion
I have now completed your drinking water test, and the test shows that the arsenic level is .... The limit of safe water in India is 10 µg/liter arsenic, more than that arsenic is considered dangerous.... µg/liter is present in your water drinking water. (show scale). It is above/below the safe limit. Water that contains any arsenic must be cleaned properly before consumption. This is the conclusion of the survey. Thank you for your time and have a nice day.
